# Castration Performed on the Patient by Himself

**Published:** 1844-02

**Authors:** 


*Castration performed on the Patient by Himself.*—An old gentleman, having suffered most excruciating pain with a scirrhous testicle, one night, when alone, was maddened to such a degree with pain that he took hold of the spermatic cord with the finger and thumb of the left hand, caught the testicle in the right hand, and commenced twisting, and he continued till he severed it. He told me himself it took fifteen rounds hard work. No hæmorrhage naturally followed, nor did any other bad symptom.—*Med. Times.*

